# Comparison of birth certificates and hospital-based birth data on pregnancy complications in Los Angeles and Orange County, California

**DOI:** 10.1186/s12884-016-0885-0

**Published:** 2016-04-27

**Authors:** Nekisa Haghighat, Maogui Hu, Olivier Laurent, Judith Chung, Peter Nguyen, Jun Wu

**Affiliations:** Program in Public Health, College of Health Sciences, University of California, Anteater Instruction & Research Bldg (AIRB) # 2034, 653 East Peltason Drive, Irvine, CA 92697-3957 USA; Maternal-Fetal Medicine, School of Medicine, University of California, Irvine, CA USA; VA Long Beach Healthcare System, Long Beach, CA USA

**Keywords:** Birth certificates, Hospital birth records, Preeclampsia, Diabetes, Socioeconomic status

## Abstract

**Background:**

The incidence of both gestational diabetes mellitus and preeclampsia is on the rise; however, these pregnancy complications may not be systematically reported. This study aimed to examine differences in reporting of preeclampsia and gestational diabetes between hospital records and birth certificate data, and to determine if such differences vary by maternal socioeconomic status indicators.

**Methods:**

We obtained over 70,000 birth records from 2001 to 2006 from the perinatal research database of the Memorial Care system, a network of four hospitals in Los Angeles and Orange Counties, California. Memorial birth records were matched to corresponding state birth certificate records and analyzed to determine differential rates of reporting of preeclampsia and diabetes. Additionally, the influence of maternal socioeconomic factors on the reported incidence of such adverse pregnancy outcomes was analyzed. Socioeconomic factors of interest included maternal education levels, race, and type of health insurance (private or public).

**Results:**

It was found that the birth certificate data significantly underreported the incidence of both preeclampsia (1.38 % vs. 3.13 %) and diabetes (1.97 % vs. 5.56 %) when compared to Memorial data. For both outcomes of interest, the degree of underreporting was significantly higher among women with lower education levels, among Hispanic women compared to Non-Hispanic White women, and among women with public health insurance.

**Conclusion:**

The Memorial Care database is a more reliable source of information than birth certificate data for analyzing the incidence of preeclampsia and diabetes among women in Los Angeles and Orange Counties, especially for subpopulations of lower socioeconomic status.

## Background

Adverse pregnancy outcomes such as gestational diabetes mellitus and preeclampsia have important consequences on the growth, development, and health of children and mothers alike. Gestational diabetes mellitus is defined as carbohydrate intolerance with onset or recognition during pregnancy [1]. The true prevalence of gestational diabetes is unknown, with estimates ranging from 1 to 14 % of pregnancies affected in the United States annually [2–4]. Preeclampsia is a pregnancy complication that is characterized by the onset of hypertension and the presence of protein in the urine at >20 weeks gestation in a previously normotensive woman [5]. It is estimated to have affected 3.8 % of US deliveries in 2010, with the rate of severe preeclampsia rapidly increasing over the past three decades [6].

Despite the fact that the incidence of both gestational diabetes and preeclampsia is on the rise, there is concern that these and other pregnancy complications are not systematically recognized, diagnosed, or reported. In fact, numerous studies have found significant inconsistency between medical information that is reported on birth certificates and other medical records such as hospital-discharge records. A recent study found that in Washington State, most pregnancy complications in general, and gestational diabetes in particular, are substantially underreported on birth certificates compared to hospital-discharge data [7]. Another study conducted in Ohio discovered that birth certificate data was less reliable than hospital records for identifying maternal risk factors, comorbid conditions, and complications of pregnancy, labor, and delivery [8]. Moreover, a number of other studies have discovered underreporting of birth defects [9], delivery complications [10], and other standard measures taken at birth [8] using state birth certificates alone. A review of the current literature suggests that birth certificates are generally not reliable sources of information on tobacco and alcohol use, prenatal care, maternal risk, pregnancy complications, labor, and delivery [11].

In addition to the question of systematic underreporting of adverse pregnancy outcomes, there is considerable interest in determining if this underreporting varies according to socioeconomic status. The socioeconomic gradient in preeclampsia is well established, with more recent data suggesting that there is a significant negative association between socioeconomic status and preeclampsia. Studies have found such an association using maternal education level [12], census tract income level [13], and household income level [5] as socioeconomic indicators. However, inconsistent findings have also been reported, with older studies finding no association between low socioeconomic status and preeclampsia [14–16].

Studies that have investigated the association between socioeconomic status and gestational diabetes, however, have generated conflicting results that vary according to the socioeconomic indicators used. Numerous studies have found low socioeconomic status to be associated with a higher risk of gestational diabetes using maternal employment status [17], neighborhood income level [18, 19], education level [20, 21], type of hospital services used [22], and family income level [23, 24] as socioeconomic indicators. In contrast, a study that measured a combination of indicators including maternal employment status, education level, parity, and monthly income level, found no association between low socioeconomic status and gestational diabetes [25]. Other studies have also found no association using neighborhood deprivation level [26], insurance status [27], and level of maternal education [28] as markers.

It has not yet been determined if the discrepancies in the results of these studies may be accounted for by differential reporting rates among populations of varying socioeconomic levels, a feature that may vary across study settings. Additionally, the reliability of California’s birth certificate registry compared to available medical records has yet to be determined. Thus, this paper examines the possibility of underreporting of adverse pregnancy outcomes in patients in Los Angeles County and Orange County, California, using data from both birth certificates and hospital birth records, with a specific focus on possible differential rates of underreporting according to socioeconomic status.

## Methods

### Birth record data

Birth record data from the period of 2001 to 2006 were obtained from the Memorial Care System, a network of four hospitals that maintains a perinatal database for research purposes [29]. Records are inputted into this database by nurses when patients are admitted to the hospital for delivery. The four hospitals – Anaheim, Long Beach, Orange Coast, and Saddleback Memorial Medical Centers – are located in Los Angeles and Orange Counties in Southern California in the United States. Additionally, birth certificate data were obtained for the four hospitals and the same study period from the California Department of Public Health [30].

### Data linkage

Birth records from the Memorial Care System were matched to birth certificate records using fuzzy matching logic through the SAS “COMPGED” function, which measures the “generalized edit distance” that summarizes the degree of difference between two text strings – i.e. the number of deletions, insertions, or replacements in the characters of a word required to arrive at the observed word [31]. Memorial records were matched to birth certificate records according to their calculated degree of similarity based on different combinations of four variables: mother’s first and last name, date of birth (DOB) of child, DOB of mother, and hospital of delivery. These variables were selected because they were the only variables available in both datasets that were specific enough to identify a particular birth. To maximize the matching rate, we conducted multiple matching procedures. Four variables were used in the matching process: DOB of child, hospital of delivery, DOB of mother, and mother’s name. The following criteria by order of priority were used to compile the final dataset: 1) An exact match on all of the four variables, 2) An exact match on DOB of child, hospital of delivery, and mother’s full name, but non-exact match on DOB of mother, 3) an exact match on DOB of child, hospital of delivery, and DOB of mother, but non-exact match on mother’s name, and 4) an exact match on DOB of child and hospital of delivery, but non-exact match on both mother’s name and DOB of mother. We assigned a partial match on DOB of mother if two of the three fields in the date of birth (i.e. year, month, day) were the same. We assumed a partial match on mother’s name if the first three characters in both the first name and the last name of the mother were matched. There are respectively ten and three records with any missing values in the four matching variables in the Memorial and the birth certificate data, and they were excluded from the matching process.

### Data analysis

We summarized the basic socio-demographic variables in both the Memorial and the birth certificate data and in the matched and the unmatched groups for each dataset. T-tests for continuous variables and chi-square tests for categorical variables were used for the comparisons of the rates of preeclampsia and diabetes, and the socio-demographic variables between the matched and the unmatched groups within each specific dataset.

Further, for the matched birth records, we compared the influence of socioeconomic factors (i.e. maternal education, race, and type of health insurance (private or public)) on the reported incidences of preeclampsia or diabetes during pregnancy. The Memorial data contained only the lumped variable of diabetes (gestational diabetes and pre-existing) during pregnancy. Prior to 2006, gestational and pre-existing diabetes were grouped into a single variable in the birth certificate data. After 2006, gestational diabetes and pre-existing diabetes were reported separately. Therefore, we analyzed diabetes over 2001–2005 and conducted sensitivity analysis using 2001–2006 data as well.

Given the potential for underreporting of adverse pregnancy outcomes on birth certificates, we did not specifically examine the agreement of the two databases using parameters such as sensitivity, specificity or Kappas. Rather, we focused on determining if underreporting of diabetes and preeclampsia would disproportionately affect low socioeconomic status groups when level of maternal education, type of health insurance, and race were used as socioeconomic indicators. In order to explore this, we calculated the ratio of incidences of diabetes (both preexisting and gestational) and preeclampsia using birth certificate data compared to Memorial data. A permutation based statistical test was used to check whether the incidence of preeclampsia and diabetes was significantly underreported on birth certificate data when compared to Memorial data. The socioeconomic variables used for the comparison were those based on birth certificate data, which have been shown to provide reliable information on these variables [32–34]. Maternal education level was categorized as either high school or lower, or college or higher. Maternal race was categorized as Asian, Black, Hispanic, Non-Hispanic White, or Other, and insurance type was defined as either public or private.

## Results

### Characteristics of study population

After removing multiple births (e.g. twins and triplicates) and the records with missing matching variables, we obtained 66,352 and 71,512 records in the Memorial and the birth certificate data, respectively. After excluding these records, a total of 62,200 records (93.7 % of Memorial data and 87.0 % of birth certificate data) were matched and subsequently used in our analysis (Table 1). The exact match on all of the four variables accounted for 95 % of all the matched records. Sensitivity analysis was conducted on the partial match on mother’s name using different lengths of the sub-string of first and last names (from left to right: 2 to 15 characters). Since the majority of records were matched by exact names, this only slightly changed the number of the final matched records (data not shown). Hence, we only reported the results based on the matching criteria listed above.

**Table 1:** Matching rates based on different matching criteria

						Number	Percentage based on Memorial Care data	Percentage based on birth certificate data
Matched records						62200	85.79 %	86.98 %
		Matching Variables						
	Types of matching	DOB of child	Delivery hospital	DOB of mother	Mother’s name			
1. Exact match on four variables		Exact	Exact	Exact	Exact	59105	81.52 %	82.65 %
2. Exact match on three variables		Exact	Exact	Partial^a^	Exact	2869	3.96 %	4.01 %
3. Exact match on three variables		Exact	Exact	Exact	Partial^b^	120	0.17 %	0.17 %
4. Exact match on two variables		Exact	Exact	Partial^a^	Partial^b^	106	0.15 %	0.15 %
Unmatched records in Memorial Care data with non-missing matching variables						4162	5.74 %	
Unmatched records in Memorial Care data with missing matching variables						6138	8.47 %	
Unmatched records in birth certificate data						9312		13.02 %

^a^matched by any two of the three fields: year, month, and day

^b^matched by the first three characters in both the first name and the last name of the mother

Summary statistics showed some differences in the characteristics of the study population between the Memorial and the birth certificate data and between the matched and unmatched records in each specific dataset (Table 2). The majority of women were between the ages of 30 and 39, had private health insurance, and had college or higher levels of education than did high school or lower (maternal education was only available on the birth certificate data). Hispanic and Non-Hispanic White made up the two largest categories in both the Memorial and the birth certificate data and in the unmatched and matched records in each dataset. Compared to the matched group, the unmatched groups on average had higher rates of preeclampsia and diabetes, were older, had lower educational attainment, and had a higher percentage of Hispanic women and a lower percentage of Non-Hispanic White and Black women in both the Memorial and the birth certificate datasets (Table 2). Since we had a large sample size (*N* = 4162 to 62200), the majority of t-tests and chi-square tests showed statistically significant results when comparing the matched and the unmatched group in each dataset. Nevertheless, the pattern of difference in the unmatched vs. matched group was consistent in the Memorial data and the birth certificate data.

**Table 2:** Characteristics of the study population from Four Memorial Care Hospitals in Southern California

	Matched memorial care data	Unmatched Memorial Care data		*p*-value^*^	Matched birth certificate data	Unmatched birth certificate data	*p*-value^**^
		Valid matching variables	Missing matching variables			Valid matching variables^a^	
N	62200	4162	6138		62200	9312	
Rate of preeclampsia (2001–2006 data)	3.13	3.73	4.12	<0.01	1.38	1.97	<0.01
Rate of preeclampsia (2001–2005 data)	3.12	3.70	4.12	<0.01	1.34	1.75	<0.01
Rate of preeclampsia (2006 data only)	3.13	3.90	N.A.	0.41	1.57	2.23	<0.01
Rate of total diabetes (2001–2005 data)	5.56	6.65	5.46	0.17	1.97	2.11	0.40
Rate of total diabetes (2006 data only)	6.93	7.20	N.A.	0.35	2.24	3.67	<0.01
Rate of gestational diabetes (2006 data only)	N.A.	N.A.	N.A.		1.62	2.57	0.01
Rate of chronic diabetes (2006 data only)	N.A.	N.A.	N.A.		0.62	1.10	0.04
Maternal age^b^ (%)				<0.01			<0.01
<20	6.62	5.77	N.A.		6.62	5.76	
20–29	42.38	39.60	N.A.		42.44	39.79	
30–39	46.75	47.57	N.A.		46.77	48.41	
40+	4.18	6.78	N.A.		4.16	6.04	
Unknown	0.07	0.29	N.A.		0.00	0.00	
Maternal age^b^ (years): mean ± standard deviation	29.79 ± 6.17	30.56 ± 6.46	N.A.	<0.01	29.78 ± 6.16	30.44 ± 6.34	<0.01
Maternal education (%)							<0.01
College or higher	N.A.	N.A.	N.A.		57.26	52.71	
High school or lower	N.A.	N.A.	N.A.		40.82	43.93	
Unknown	N.A.	N.A.	N.A.		1.92	3.36	
Race/ethnicity^b^ (%)				<0.01			<0.01
Asian	10.49	9.61	N.A.		13.65	14.85	
Black	8.47	6.68	N.A.		8.96	6.20	
Hispanic	33.01	42.74	N.A.		36.97	39.42	
Non-Hispanic White	39.62	30.56	N.A.		37.62	35.72	
Other	4.52	5.00	N.A.		1.91	1.98	
Unknown	3.88	5.41	N.A.		0.89	1.84	
Insurance^b^ (%)				<0.01			<0.01
Private	65.95	57.81	N.A.		66.77	66.91	
Public	29.97	35.66	N.A.		32.69	32.25	
Other	0.00	0.00	N.A.		0.44	0.63	
Unknown	4.07	6.54	N.A.		0.11	0.20	

^*^ *p*-value for the comparison between the matched Memorial Care and the unmatched Memorial Care data with valid matching variables

^**^ *p*-value for the comparison between the matched birth certificate data and the unmatched birth certificate data (all had valid matching variables)

^a^All records in the unmatched birth certificate data had valid matching variables

^b^Both databases have reported values for these variables. In this table, we calculated the statistic summaries based on the values reported in each specific database

### Preeclampsia: 2001–2006

It was found that the birth certificate data significantly underreported the incidence of preeclampsia when compared to the Memorial data (1.38 % vs. 3.13 %) (Table 2) (*p* < 0.01). The underreporting in birth certificate data was also observed in subcategories of the socioeconomic variables we examined.

#### Percentage of preeclampsia by maternal education

Of the mothers with known education level, the birth certificate data showed that the incidence of preeclampsia was significantly higher among mothers with education levels of college or higher compared with mothers with education levels of high school or lower (1.46 % vs. 1.24 %; *p* = 0.02) (Table 3). No difference was observed in the Memorial data. Nevertheless, the degree of underreporting of preeclampsia using birth certificate data compared to Memorial data was significantly higher among women with lower education levels (*p* = 0.01).

**Table 3:** Incidence rate of preeclampsia (2001–2006) by socioeconomic indicators

		Number	Incidence rate: Memorial data	Incidence rate: birth certificate data	Ratio of incidence rate: birth certificate/Memorial data
Maternal Education^a^	College or higher	35616	3.11 %	1.46 %	0.47
	High School or lower	25389	3.17 %	1.24 %	0.39
	Unknown	1195	2.76 %	2.01 %	0.73
	*p*-value: College or higher vs. High school or lower		0.65	0.02*	0.01*
Race/ethnicity^a^	Asian	8489	2.67 %	1.06 %	0.40
	Black	5576	4.12 %	1.90 %	0.46
	Hispanic	22995	3.20 %	1.25 %	0.39
	Non-Hispanic White	23400	2.95 %	1.48 %	0.50
	Other	1187	4.13 %	1.52 %	0.37
	Unknown	553	2.35 %	1.81 %	0.77
	*p* -value: Asian-Black		0.00*	0.00*	0.20
	*p* -value: Asian-Hispanic		0.01*	0.15	0.48
	*p* -value: Black-Hispanic		0.00*	0.00*	0.15
	*p* -value: Hispanic- NH White		0.12	0.03*	0.01*
Insurance^a^	Private	41024	3.02 %	1.46 %	0.48
	Public	18643	3.32 %	1.30 %	0.39
	Other	2527	3.44 %	0.79 %	0.23
	Unknown	6	0.00 %	0.00 %	N.A.
	*p*-value: Private vs. Public		0.05	0.12	0.02*

^a^The classification of socioeconomic status was based on variables reported in the birth certificate data

*Statistically significant result at an alpha level of 0.05

#### Percentage of preeclampsia by race

Both the Memorial and birth certificate data indicated the highest rate of preeclampsia in Black women (4.12 and 1.90 %, respectively) among the four major race/ethnicity categories. Within the Memorial data, Asian and Hispanic women had significantly lower rates of preeclampsia than Black women (*p* < 0.01). The same held true for the birth certificate data. In addition, Hispanic women had significantly lower rates of preeclampsia than Non-Hispanic White women (*p* = 0.03) in the birth certificate data, while Asian women had significantly lower rates of preeclampsia than Non-Hispanic White women (*p* = 0.01) in the Memorial data.

Compared to the Memorial data, the birth certificate data showed a significantly higher degree of underreporting of preeclampsia in Hispanic women compared to Non-Hispanic White women (*p* = 0.01). No significant difference for the degree of underreporting in birth certificate data was observed between the other race/ethnicity groups.

#### Percentage of preeclampsia by insurance

The Memorial data indicated a marginally significantly lower rate of preeclampsia in women with private insurance compared to those with public insurance (3.32 % vs. 3.02 %; *p* = 0.05). A different but insignificant pattern was observed in the birth certificate data. Compared to the Memorial data, the birth certificate data showed a significantly higher degree of underreporting of preeclampsia within the public insurance group compared to the private insurance group (*p* = 0.02).

### Diabetes (2001–2005)

Similar to preeclampsia, the birth certificate data significantly underreported the incidence of diabetes when compared to the Memorial data (1.97 % vs. 5.56 %) (Table 2) (*p* < 0.01). Memorial data indicated that the incidence rate of diabetes during pregnancy was higher among women with lower socioeconomic status, but the pattern was insignificant in the birth certificate data. Sensitivity analysis showed similar results based on data in the 2001–2006 period (results not shown).

#### Percentage of diabetes by maternal education

No significant patterns were observed in birth certificate data. However, of the women whose educational level was known, the Memorial data indicated that the incidence of diabetes was significantly higher among women with levels of education of high school or lower compared to college or higher (6.18 % vs. 5.18 %; *p* < 0.01) (Table 4). The birth certificate data showed a similar but insignificant pattern. Furthermore, the degree of underreporting of diabetes using birth certificate data compared to memorial data was significantly higher among women with lower education levels (*p* = 0.01).

**Table 4:** Incidence rate of diabetes (2001–2005) by socioeconomic indicators

		Number	Incidence rate: Memorial data	Incidence rate: birth certificate data	Ratio of incidence rate: birth certificate/Memorial data
Maternal Education^a^	College or higher	29653	5.18 %	1.95 %	0.38
	High School or lower	20917	6.18 %	2.01 %	0.32
	Unknown	973	3.49 %	1.75 %	0.50
	*p*-value: College or higher vs. High school or lower		0.00*	0.66	0.01*
Race/ethnicity^a^	Asian	6897	7.41 %	2.65 %	0.36
	Black	4561	4.93 %	1.67 %	0.34
	Hispanic	18755	6.95 %	2.24 %	0.32
	Non-Hispanic White	19856	3.63 %	1.54 %	0.42
	Other	965	8.19 %	2.38 %	0.29
	Unknown	509	4.52 %	1.77 %	0.39
	*p* -value: Asian-Black		0.00*	0.00*	0.35
	*p* -value: Asian-Hispanic		0.21	0.06	0.15
	*p* -value: Black-Hispanic		0.00*	0.01*	0.37
	*p* -value: Hispanic- NH White		0.00*	0.00*	0.00*
Insurance^a^	Private	34489	5.25 %	1.95 %	0.37
	Public	15034	6.25 %	2.02 %	0.32
	Other	2015	5.56 %	1.94 %	0.35
	Unknown	5	0.00 %	0.00 %	N.A.
	*p*-value: Private vs. Public		0.00*	0.61	0.05*

^a^The classification of socioeconomic status was based on variables reported in the birth certificate data

*Statistically significant result at an alpha level of 0.05

#### Percentage of diabetes by race

The birth certificate data found that the incidence of diabetes was highest among Asian women (2.65 %) and lowest among Non-Hispanic White women (1.54 %), while the Memorial data indicated the highest incidence rate among women of race/ethnicities classified as “other” (8.19 %) and Asian (7.41 %) and lowest among Non-Hispanic White women (3.63 %). For both the Memorial and birth certificate data, the difference in incidence rates was significant (*p* < 0.01) between all the race/ethnicity group pairs except Asian and Hispanics. Furthermore, the degree of underreporting using birth certificate data compared to Memorial data was significantly higher among Hispanic women compared to Non-Hispanic White women (*p* < 0.01).

#### Percentage of diabetes by insurance

Memorial data found that the incidence of diabetes was significantly higher among women with public health insurance compared to women with private health insurance (6.25 % vs. 5.25 %, *p* < 0.01). The birth certificate data showed a similar but insignificant pattern. The degree of underreporting of diabetes using birth certificate data compared to Memorial data was significantly higher among women with public insurance compared to those with private insurance (*p* = 0.05).

## Discussion

We found that the birth certificate data that was analyzed significantly underreported the incidences of preeclampsia and diabetes compared to the Memorial data, which was collected specifically for research purposes. In addition, the degree of underreporting was disproportionately distributed across groups of different socioeconomic status, with certain socioeconomic indicators exhibiting higher degrees of underreporting.

The degree of underreporting of both preeclampsia and diabetes using birth certificate data was significantly higher women with lower education levels compared to women with higher levels of education, in Hispanic women compared to non-Hispanic White women, in women with public insurance compared to those with private insurance. These results indicate a disparate underreporting problem in low socioeconomic groups for pregnancy complications when race, level of education, and insurance status are used as socioeconomic indicators.

Several reasons may exist to explain the discrepancies between our two datasets, such as the weaknesses of birth certificate data discovered by several other studies. These include inadequate auditing of birth certificate data by individual hospitals, variations in data collection, diagnosis, and reporting procedures across hospitals, the use of nonclinical or untrained personnel to record data, and budgetary restrictions that prevent state agencies from thoroughly assessing and ensuring the quality of birth certificate data [8, 10, 35–38]. The Memorial data, in turn, may have had better quality on pregnancy complications because it was a research database that underwent more stringent quality checks by nurses. Although the Memorial database is not a gold standard, it is believed that it is more accurate than birth certificate data because information is recorded when patients are physically present and able to verify records.

We attempted to maximally match the Memorial and birth certificate records. However, there were still 8.47 % and 5.74 % of the Memorial records that could not be matched to the birth certificates due to missing matching variables (i.e. mother’s name and mother’s date of birth) and likely moderate to serious problems in misspelling of the names, respectively. We found differences in the matched and unmatched groups in the rates of preeclampsia and diabetes and in socio-demographic parameters. But since the patterns of difference in the matched and the unmatched groups was consistent between the Memorial data and the birth certificate data, we do not expect it to change our main conclusion of the underreporting problem in the birth certificate data.

Since the Memorial data did not differentiate between gestational diabetes and diabetes when reporting pregnancy outcomes, it was not possible to investigate gestational diabetes specifically in this study. However, because the birth certificate data began reporting gestational diabetes separately starting in 2006, we were able to perform two separate analyses on diabetes using 2001–2005 and 2001–2006 as periods of interest. Both periods showed the same patterns of underreporting of diabetes, and we thus suspect that our findings on total diabetes likely hold for gestational diabetes as well. It has been estimated that approximately 90 % of pregnancies that are complicated by diabetes mellitus represent women with gestational diabetes mellitus [39]. In the 2006 birth certificate data in California, we observed that gestational diabetes accounted for 72 % of the total diabetes (Table 2). This high degree of overlap between gestational diabetes and diabetes during pregnancy further suggests that the findings of this study regarding diabetes in general may also be applicable to cases of gestational diabetes in particular.

These results are consistent with previous studies, particularly those that determined that birth certificates are not reliable sources of information regarding preeclampsia, gestational diabetes, and other maternal complications and characteristics, particularly when compared to hospital discharge records [7–11]. Thus, our conclusion that the birth certificate database used in this study underreported the incidence of preeclampsia and gestational diabetes is supported by similar patterns found elsewhere in the United States. However, to our knowledge this is the first study to assess the reliability of hospital data and birth certificates in southern California, and the first to address differential reporting of preeclampsia and diabetes during pregnancy by socioeconomic status in the United States.

The socioeconomic differences seen in the underreporting of preeclampsia and gestational diabetes as specific outcomes of interest is a unique observation that has not been studied in southern California. However, similar results have been found by studies that have analyzed related, though not identical, variables elsewhere in the United States. Most notably, a study on the sensitivity of birth certificate reports of birth defects in Atlanta found that Non-Hispanic Black maternal race/ethnicity and maternal education levels lower than high school were independently associated with a lower probability of a birth defect diagnosis being reported on a birth certificate [9]. The authors of this study hypothesized that this observation might be explained by disparities in access to healthcare, as well as variations in personnel and birth certificate completion procedures across hospitals. Although our study did not analyze birth defects, the underreporting of adverse pregnancy outcomes we found according to racial and education level factors followed a similar pattern and can be explained by the same observations.

Further research must be performed to elucidate an explanation for the poor reliability of this particular set of birth certificate data for pregnancy complications, as well as the observed socioeconomic gradient in underreporting of such outcomes. Nevertheless, these findings have important implications for future public health research. Studies that rely solely on birth certificate data to draw conclusions regarding pregnancy complications should be aware of a potential bias towards underestimating the incidence of these conditions, particularly in low socioeconomic groups. This is critical for the descriptive study of socioeconomic disparities in pregnancy complications, and might contribute to explain why discrepant results were reported in the past [17–28], beside any true difference in disparities across study settings. Such biases are also critical for etiologic research studying the relationships between pregnancy complications and potential risk factors, especially when these are unevenly distributed according to socioeconomic status. For instance, exposure to most air pollutants (e.g., primary particles from road traffic) is typically higher in populations with low socioeconomic status than in better-off ones [40, 41]. In such a situation, a higher underreporting of maternal complications in populations with lower socioeconomic status would create a downward bias while measuring the association between air pollution and pregnancy complications. Consequently, researchers should attempt to use high quality health outcome data such as the Memorial database, either in place of or in conjunction with birth certificate data, whenever possible in order to minimize bias.

Furthermore, these findings indicate that there is a considerable need to improve the quality of birth certificate data in California, as far as pregnancy complications are concerned. There is a possibility that the quality of birth certificate has improved since 2006, the last year of this analysis. It would be beneficial to assess the quality of current birth certificate data in order to identify areas that still require improvement. However, historical birth certificate data are still of high importance for research studies that examine the impact of *in-utero* exposure on various long-term health effects (e.g. cognition and school performance in children, obesity, cardiovascular diseases etc.). Standardizing data collection and reporting procedures across hospitals would help minimize the discrepancies seen between birth certificate data and hospital databases such as the Memorial database. Because diabetes and preeclampsia are conditions that are oftentimes diagnosed prior to delivery and not at the hospital of delivery, there is also a need to improve the integration of prior medical records from other sources with hospital and birth certificate records. What is more, the fact that the birth certificate data underreported both preeclampsia and diabetes and did so to a higher degree among groups of lower socioeconomic status suggests that it would be most effective to focus standardization efforts on these particular conditions and among these identified groups, including Black and Hispanic women, women with lower levels of education, and women with public insurance. Finally, the most disadvantaged women may not have access to health care; thus improving health care access for low-income and minority people may also improve the reporting of pregnancy complications.

## Conclusion

In summary, this comparison of two birth record databases found that the Memorial database is a more reliable source of information than birth certificate data for analyzing the incidence of preeclampsia and gestational diabetes among women in Los Angeles County. This is especially true for subpopulations of lower socioeconomic status. Efforts to improve the available sources of data for the study of adverse pregnancy outcomes should thus focus on improving the reliability of birth certificate data, particularly for women of lower socioeconomic status.

## Declarations

### Ethics and consent

The study has been approved by the Institutional Review Board of the University of California, Irvine and by the Institutional Review Board of MemorialCare Health System and Long Beach Memorial Medical Center. However, informed consent from study participants was not required because the nature of the study was analysis of existing data, which posed minimal risk to the subjects. In addition, it was not practically feasible to contact all the subjects.

### Consent to publish

Not applicable.

### Availability of data and materials

The birth record data used in this study will not be shared because they contained confidential information including the name of the mother and the date of birth of both the mother and the infant.

## Abbreviation

DOB: date of birth
